# Enhanced recovery after robotic ventral hernia repair: factors associated with overnight stay in hospital

**DOI:** 10.1007/s10029-023-02871-3

**Published:** 2023-09-05

**Authors:** M. Marckmann, P.-M. Krarup, N. A. Henriksen, M. W. Christoffersen, K. K. Jensen

**Affiliations:** 1 Digestive Disease Center, Bispebjerg Hospital, University of Copenhagen, Bispebjerg Bakke 23, 2400 Copenhagen, Denmark; 2 Department of hepatic and gastrointestinal diseases, Herlev Hospital, University of Copenhagen, Copenhagen, Denmark; 3 Department of Surgery and Transplantation, Rigshospitalet, Copenhagen University Hospital, Copenhagen, Denmark

**Keywords:** ERAS, Robot-assisted surgery, Ventral hernia repair, Length of stay

## Abstract

**Purpose:**

Enhanced recovery after surgery (ERAS) protocols lead to reduced post-operative stay and improved outcomes after most types of abdominal surgery. Little is known about the optimal post-operative protocol after robotic ventral hernia repair (RVHR), including the potential limits of outpatient surgery. We report the results of an ERAS protocol after RVHR aiming to identify factors associated with overnight stay in hospital, as well as patient-reported pain levels in the immediate post-operative period.

**Methods:**

This was a prospective cohort study of consecutive patients undergoing RVHR. Patients were included in a prospective database, registering patient characteristics, operative details, pain and fatigue during the first 3 post-operative days and pre- and 30-day post-operative hernia-related quality of life, using the EuraHS questionnaire.

**Results:**

A total of 109 patients were included, of which 66 (61%) underwent incisional hernia repair. The most performed procedure was TARUP (robotic transabdominal retromuscular umbilical prosthetic hernia repair) (60.6%) followed by bilateral roboTAR (robotic transversus abdominis release) (19.3%). The mean horizontal fascial defect was 4.8 cm, and the mean duration of surgery was 141 min. In total, 78 (71.6%) patients were discharged on the day of surgery, and factors associated with overnight stay were increasing fascial defect area, longer duration of surgery, and transverse abdominis release. There was no association between post-operative pain and overnight hospital stay. The mean EuraHS score decreased significantly from 38.4 to 6.4 (*P* < 0.001).

**Conclusion:**

An ERAS protocol after RVHR was associated with a high rate of outpatient procedures with low patient-reported pain levels.

## Introduction

Robotic approach for ventral hernia repair is a rapidly emerging approach with promising results in recent years compared to open standard approaches [1–6]. Since the introduction, surgeons worldwide are using the robotic approach for an increasing amount of abdominal wall procedures and the indication for robotic repair, thus, widens. One key advantage of robotic ventral hernia repair seems to be a reduced length of post-operative hospital stay, which can be compared to the enhanced recovery after various other surgical procedures when traditional open surgery was replaced by minimally invasive approaches [7].

Enhanced recovery after surgery (ERAS) protocols improve post-operative outcomes after most types of abdominal surgery [8, 9]. In recent years, enhanced recovery after open abdominal wall reconstruction procedures has reportedly decreased length of stay with no change in the incidence of readmissions or post-operative complications [10, 11]. However, little is known about the optimal post-operative protocol after robotic ventral hernia repair, including the potential limits of outpatient surgery.

In the current study, we report the results of an ERAS protocol after robotic ventral hernia repair, with the aim to identify factors associated with overnight stay in the hospital, as well as patient-reported pain levels in the immediate post-operative period.

## Methods

This was a prospective single-center cohort study including consecutive patients undergoing robotic ventral hernia repair at a university hospital in Copenhagen, Denmark. All patients were seen in the outpatient clinic and underwent computed tomography scan as part of the pre-operative planning.

### ERAS pathway

**Fig. 1:**
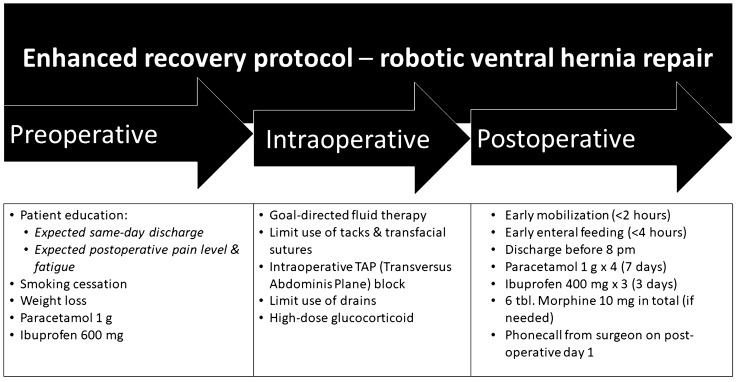
Enhanced recovery protocol after robot-assisted ventral hernia repair

The ERAS protocol used in the current study was built on evidence-based measures to improve post-operative recovery of patients after abdominal surgery [12]. The main concepts of the protocol are summarized in Fig. 1. Pre-operatively, patients were educated on expected same-day discharge as well as the expected post-operative levels of pain and fatigue. Smoking cessation was desired; however, patients with recurring hernia incarceration or severe pain were allowed to undergo surgery without smoking cessation. Similarly, weight loss to body mass index < 35 kg/m^2^ was desirable but not mandatory. On the morning of surgery, patients were given 1 g of paracetamol and 600 mg ibuprofen. Intraoperatively, goal-directed fluid therapy was used, and high-dose glucocorticoid (Solu-Medrol 125 mg iv) was administered [13]. Urinary catheter was only applied in case of surgery duration above 3 h, and if so, removed immediately after surgery. Post-operatively, patients were mobilized within 2 h and started early enteral feeding. The expected discharge was at latest 8 pm on the day of surgery and patients were given paracetamol 1 g × 4 and ibuprofen 400 mg × 3 for seven and three days, respectively. In addition, a total of six tablets of 10 mg morphine were given to the patient, with instructions to only use if neccesitated by severe pain. Discharge criteria for patients were: (1) ability to dress independently, (2) ability to manage personal hygiene, (3) acceptable pain level (< 5 on a 10 level Numeric Rating Scale during activity), (4) patient acceptance of discharge, (5) oral food and liquid intake, and 6) transcutaneous saturation > 0.92 without oxygen supplement. The surgical procedures were performed in accordance with what was previously described in detail [14].

### Data collection

Pre-operatively registered variables included age, sex, American Society of Anesthesiologists (ASA) score, smoking status, body mass index (BMI), and type of ventral hernia (primary/incisional). Data collected intra- and post-operatively were fascial defect size, duration of surgery, type of procedure performed, and complications within 30 days post-operatively graded by the Clavien–Dindo score.

### Outcomes

The primary outcome was length of post-operative hospital stay, categorized as 0 or > 0 days. Secondary outcomes included hospital readmission and post-operative complications, defined as any deviation from the standard post-operative course within the first 30 post-operative days. Patient-reported hernia-related quality of life was assessed using the EuraHS questionnaire and registered pre-operatively on the day of surgery and 30 days (± 3 days) post-operatively. In addition, patient-reported fatigue as well as pain in supine position and during movement were registered using a numerical rating scale from 0 to 10 and repeated throughout the first three post-operative days at 8 am and 8 pm each day.

### Statistics

Patients who were discharged on the day of surgery were compared with those requiring overnight or longer stays. Categorical variables were shown as n (%) and compared across groups using the Chi-square test. Numerical variables were shown as mean (standard deviation (SD)) or median (interquartile range (IQR)) and compared across the two groups using Student’s *t* test or or Mann Whitney *U* test. In a subgroup analysis using similar statistics, only patients undergoing a TAR procedure were included. *P* values < 0.05 were considered statistically significant. Statistical analyses were performed using R version 4.2.2 (R Foundation for Statistical Computing, Vienna, Austria). The study was approved by the Regional Data Protection Agency of the Capitol Region of Denmark (ref. P-2021-58) and patients gave written consent to inclusion in the study at the time of operative planning.

## Results

In the period from March 4, 2021, to December 22, 2022, a total number of 109 patients underwent robotic ventral hernia repair at our institution. Overall, 78 (71.6%) were discharged on the day of surgery whereas the rest required overnight stay with a median (IQR) length of stay (LOS) of 1 (1) day. The two groups were comparable regarding age, BMI, ASA score, and smoking status including insignificant differences of 24 (30%) patients with BMI < 35 kg/m^2^ requiring overnight stay vs. 7 (24.1%) with a BMI > 35 (*P* = 0.719), and 7 (19.4%) patients actively smoking vs. 24 (32.9%) non-smokers requiring overnight stay (*P* = 0.216). In the group of patients discharged the same day, there were more males compared to the group of patients with overnight stay (52 (66.7%) vs. 13 (41.9%), *P* = 0.031).

Overall, 66 (60.6%) and 43 (39.4%) of all the patients underwent incisional hernia repair and umbilical or epigastric hernia repair, respectively. There were significantly more patients with incisional hernia that stayed overnight compared with primary ventral hernia repair (26 (83.9%) vs. 40 (51.3%), *P* = 0.003). The mean horizontal fascial defect was 4.8 cm with significant smaller defects in the group with same-day discharge, which was also the case regarding vertical defect size and total defect area (*P* < 0.001 for all three parameters, see Table 1). Mean duration of surgery was 141 (± 97.2) min with longer operating times for patients who stayed overnight (185 ± 90.6 min vs. 123.3 ± 94.7 min, *P* = 0.002).

**Table 1:** Baseline data comparing patients that stayed in hospital overnight with patients who were discharged same day of surgery after robotic ventral hernia repair

	Overnight stay [*n* = 31]	Same-day discharge [*n* = 78]	*P*
Age			
Mean (SD)	62.2 (14.3)	57.6 (12.4)	0.069
Sex			
Female	18 (58.1)	26 (33.3)	0.031
ASA			
I	3 (9.7)	10 (12.8)	0.333
II	17 (54.8)	51 (65.4)	
III	11 (35.5)	17 (21.8)	
Body mass index, kg/m^2^			
Median (range)	30 (18.4;46.1)	31 (21.7;44.6)	0.528
Current smoker	7 (22.6)	29 (37.2)	0.216
Non-smoker	24 (77.4)	49 (62.8)	
Type of hernia			
Incisional	26 (83.9)	40 (51.3)	0.003
Primary ventral	5 (16.1)	38 (48.7)	
Vertical defect, centimeters			
Mean (SD)	9.2 (6.4)	5.6 (4.5)	< 0.001
Horizontal defect, centimeters			
Mean (SD)	6.4 (3.7)	4.2 (2.7)	< 0.001
Area of defect, centimeters			
Mean (SD)	77.5 (82.3)	33.1 (53.4)	< 0.001
Surgery duration, minutes			
Mean (SD)	185 (90.6)	123.3 (94.7)	0.002
Mesh dimensions, centimeters			
Mean (SD)	433.4 (298.4)	252.0 (173.0)	< 0.001
Clavien–Dindo score			
0	25 (80.6)	73 (93.6)	0.071
I	6 (19.4)	4 (5.1)	
3B	0	1 (1.3)	
Type of procedure			
TAPP	2 (6.5)	5 (6.4)	0.002
RoboTAR	13 (41.9)	8 (10.3)	
TARUP	16 (51.6)	55 (70.5)	
eTEP	0 (0.0)	9 (11.5)	
Retromuscular	0 (0.0)	1 (1.3)	
Transversus abdominis release			
TAR	15 (48.4)	15 (19.2)	0.005
Type of TAR			
None	16 (51.6)	63 (80.8)	< 0.001
Bilateral	12 (38.7)	7 (9.0)	
Unilateral	3 (9.7)	8 (10.2)	
Length of stay, days			
Median (IQR)	1.0 (1)	0 (0)	< 0.001
Readmission	2 (6.5)	2 (2.6)	0.682

*SD* standard deviation, *IQR* Interquartile range, *ASA* American Society of Anesthesiologists score, *TAPP* transabdominal preperitoneal prosthesis repair, *RoboTAR* robotic transversus abdominis release, *TARUP* robotic transabdominal retromuscular umbilical prosthetic hernia repair, *eTEP* extended totally extraperitoneal repair

The most performed procedure was robotic transabdominal retromuscular umbilical prosthetic hernia repair (TARUP) (65.1%) and there was a significant difference in the type of procedure performed when comparing patients discharged the same day and patients staying overnight. Robotic transversus abdominis release (RoboTAR) was performed in 13 cases (41.9%) of the patients staying overnight compared to 8 (10.8%) in the group of patients discharged the same day (*P* = 0.002). In total, 27.5% of all the patients underwent either a unilateral or bilateral TAR procedure. Patients undergoing a TAR procedure accounted for nearly 50% of the cases who stayed overnight compared to approximately 20% in the same-day surgery group yielding a significant difference (15 (48.4%) vs. 15 (19.2%), *P* = 0.005).

Finally, 94 (86.2%) of the patients experienced no post-operative complications and there was no significant difference between the two groups with referral to Clavien–Dindo score (*P* = 0.071). One patient required operative reintervention on day five due to complete rupture of both posterior and anterior rectus sheath, resulting in small bowel obstruction. The patient underwent emergency laparotomy with suture of both layers and onlay mesh repair, without need for bowel resection, and recovered uneventfully. A total number of four (3.7%) patients were readmitted during the first 30 post-operative days, and there was no statistically significant difference in these numbers when comparing the two groups (6.5% vs. 2.6%, *P* = 0.682).

**Fig. 2:**
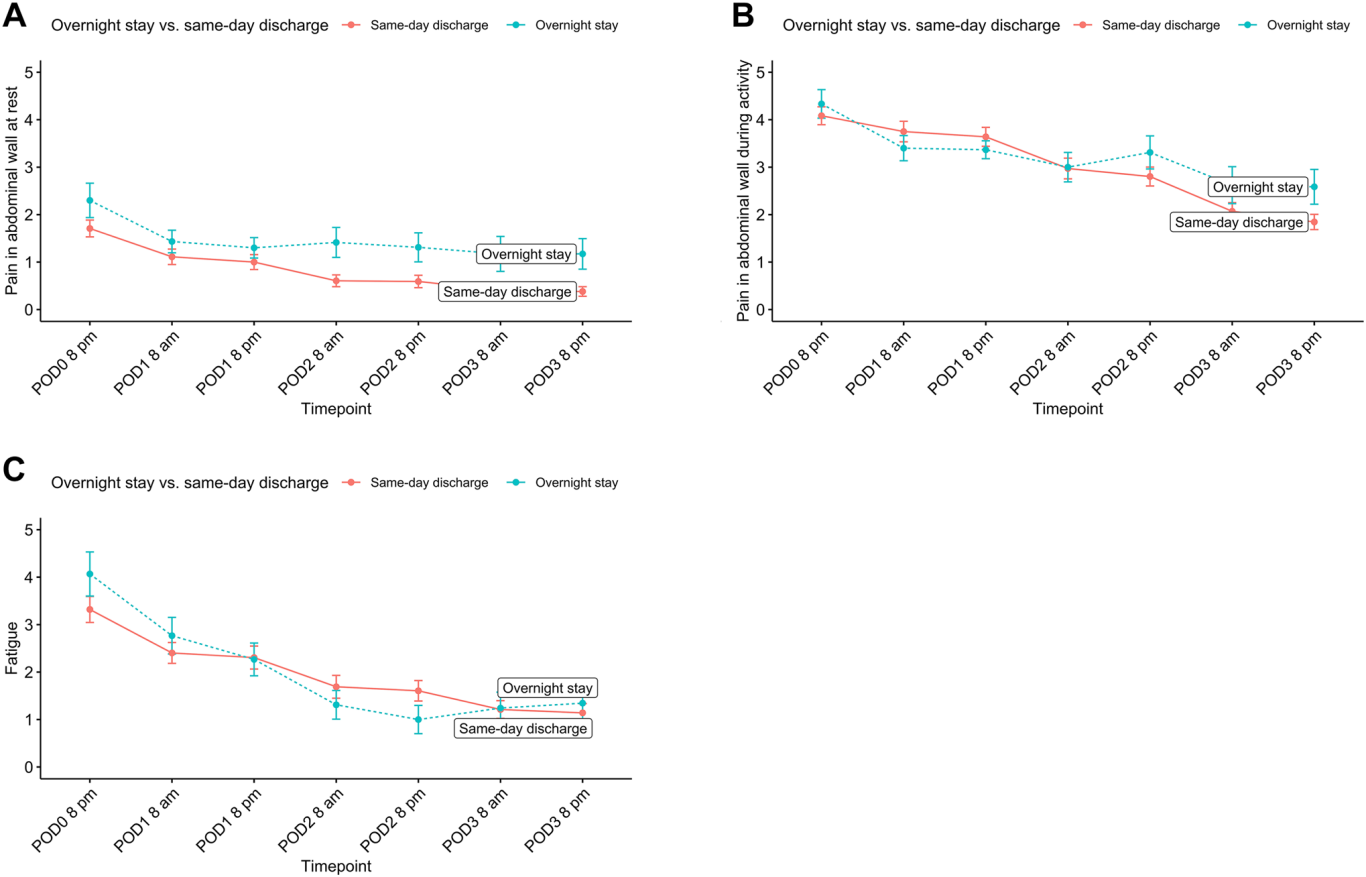
Patient-reported pain in abdominal wall at rest (**A**), during activity (**B**), and patient-reported fatigue (**C**) the first three days after robotic ventral hernia repair stratified by overnight stay vs. same-day discharge (*POD* post-operative day)

**Fig. 3:**
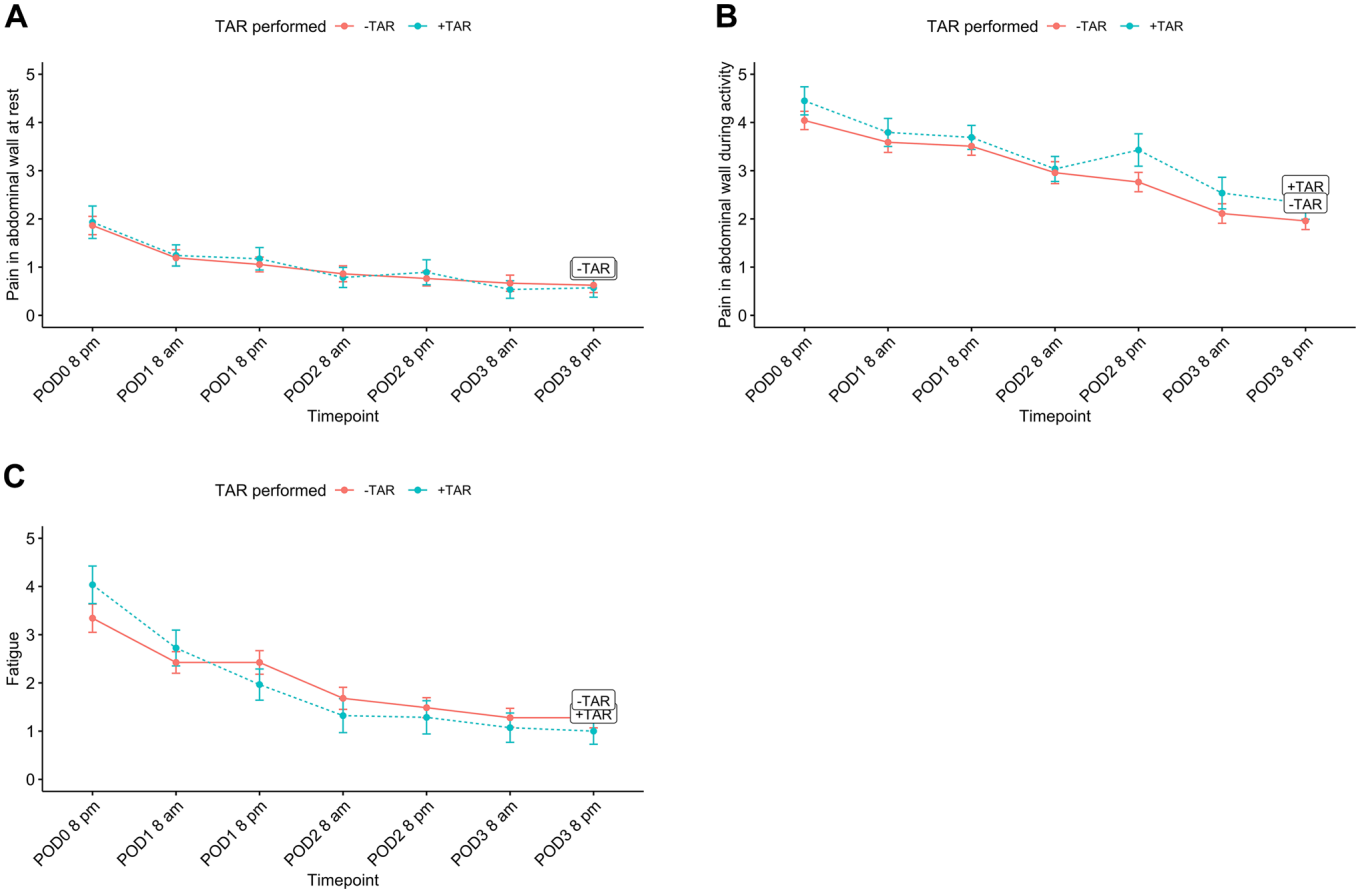
Patient-reported pain in abdominal wall at rest (**A**), during activity (**B**), and patient-reported fatigue (**C**) the first three days after robotic ventral hernia repair stratified by transversus abdominis release (TAR) procedure (*POD* post-operative day)

**Fig. 4:**
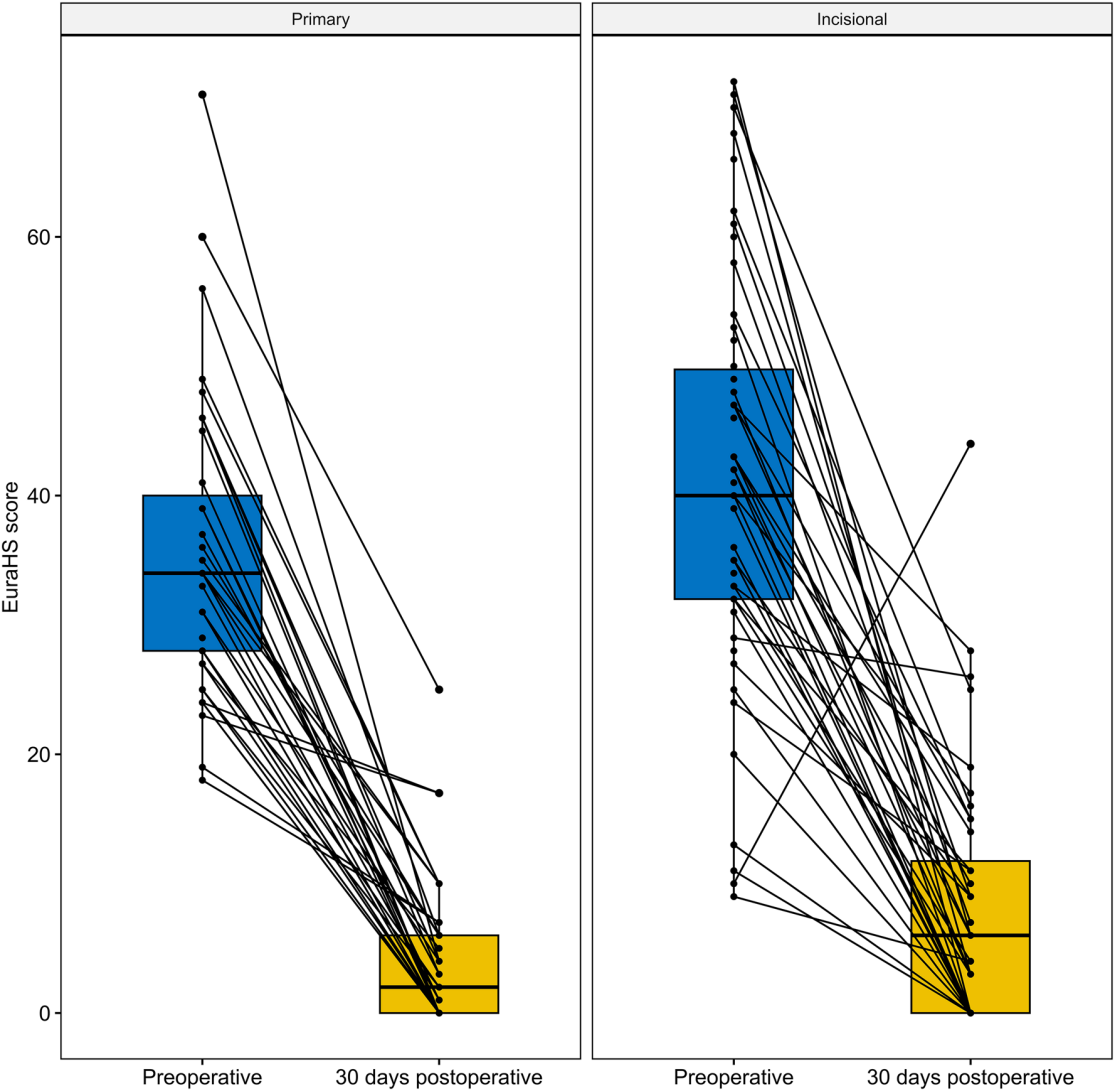
Hernia-related quality of life before and 30 days after robot-assisted ventral hernia repair measured by the EuraHS score

The patient-reported pain and fatigue during the first three post-operative days is shown in Fig. 2. The mean patient-reported levels of pain at rest and during activity on the evening of surgery were comparable between patients discharged the same day and those requiring overnight stay (1.4 vs. 1.1, *P* = 0.275) and (3.4 vs. 3.8, *P* = 0.353), respectively. The mean level of fatigue was also comparable between the two groups (2.8 vs. 2.4, *P* = 0.387). Figure 3 illustrates pain and fatigue stratified by TAR procedure (yes/no). The patient-reported EuraHS score for all patients decreased significantly at 30-day follow-up (mean 38.4 ± 13.9 vs. 6.4 ± 8.3, P < 0.001). EuraHS scores stratified by type of hernia are shown in Fig. 4.

Subgroup analysis on patients who all underwent a TAR procedure, including a horizontal defect size of > 7 cm, showed that 50% of these required overnight stay. In this group, RoboTAR procedure accounted for 13 (86.7%) vs. 8 (53.3%) in the group of patients with same-day discharge; however, this finding was not statistically significant (*P* = 0.111), see Table 2.

**Table 2:** Subgroup analysis of patients who underwent *transversus abdominis release* comparing same-day discharge with those requiring overnight stay

	Overnight stay [*n* = 15]	Same-day discharge [*n* = 15]	*P*
Age			
Mean (SD)	61.3 (14.7)	60.9 (12.6)	0.936
Sex			
Female	7 (46.7)	10 (66.7)	0.461
ASA			
I	1 (6.7)	0 (0.0)	0.357
II	10 (66.7)	13 (86.7)	
III	4 (26.6)	2 (13.3)	
Body mass index, kg/m^2^			
Median (range)	28 (21.6;46.1)	29.7 (21.7;38.3)	0.419
Smoking			
Current	4 (26.7)	5 (33.3)	0.656
Never	4 (26.7)	2 (13.3)	
Previous	7 (46.6)	8 (53.3)	
Type of hernia			
Incisional	14 (93.3)	14 (93.3)	1.000
Primary ventral	1 (6.7)	1 (6.7)	
Vertical defect, centimeters			
Mean (SD)	14 (5.7)	11.9 (6.2)	0.324
Horizontal defect			
Mean (SD)	9.5 (2.8)	8.6 (3.2)	0.410
Area of defect, centimeters			
Mean (SD)	140.7 (77.5)	113.5 (81.0)	0.347
Surgery duration, minutes			
Mean (SD)	262.7 (55.7)	229.3 (170.3)	0.470
Mesh area, centimeters			
Mean (SD)	689.1 (220.0)	477.9 (249.7)	0.014
Clavien–Dindo score			
0	13 (86.7)	15 (100.0)	0.464
I	2 (13.3)	0 (0.0)	
Type of procedure			
RoboTAR	13 (86.7)	8 (53.3)	0.111
TARUP	2 (13.3)	7 (46.7)	
Length of stay, days			
Median (IQR)	1.0 (2)	0 (0.0)	< 0.001
Readmission	1 (6.7)	0 (0.0)	> 0.99

*SD* standard deviation, *IQR* interquartile range, *ASA* American Society of Anesthesiologists score, *RoboTAR* robotic transversus abdominis release, *TARUP* robotic transabdominal retromuscular umbilical prosthetic hernia repair

## Discussion

Same-day discharge is possible in patients undergoing robotic ventral hernia repair with the use of an ERAS protocol. Incisional hernia repairs, the use of a TAR procedure, and larger horizontal defect size were associated with overnight stay in hospital.

The mean length-of-stay was 1.6 days in the current study, which is slightly lower than other studies examining outcomes for robotic hernia repair, all of which did not report to follow an ERAS protocol [15–19]. The use of an ERAS pathway in the current study may explain this difference, as supported by other studies’ findings of shorter LOS in open hernia repair comparing ERAS groups with non-ERAS groups [10]. A single retrospective study on robotic hernia repair had a similar same-day discharge rate, but this solely included cases who underwent TAPP inguinal hernia repair [20]. Local traditions and different reimbursement systems may also explain reasons for longer stay in the hospital in some countries.

To our knowledge, this is the first study on robotic ventral hernia repair in an ERAS protocolled setting. The literature on predictors of length of stay for patients undergoing non-robotic surgery is limited. Akinci et al. retrospectively examined 1170 hernia repairs from 2005 to 2010 and found increasing length of procedure as an independent predictive risk factor for longer hospital stay, echoing our findings [21]. The same study reported type of repair as a significant predictor, but only for primary ventral hernias, and not inguinal or incisional. Our results pointed to the same association regarding type of repair; however, the procedures involved in the current study were of other types, namely since our study setting exclusively considered robotic approaches with newer retromuscular techniques and TAR included. In a previous cohort of patients undergoing open incisional hernia repair at our institution, pain and lack of bowel function were found to be causes of prolonged LOS, not comparable to the current study [22].

In accordance with previously published results, we found that most of the patients who stayed overnight underwent incisional hernia repair reflecting the higher complexity of these procedures compared to primary ventral hernia repair [23].

Female sex has been reported to be associated with increased LOS after laparoscopic ventral hernia repair, an association we also found [24]. We do, however, hypothesize that this association is merely coincidental, and to a higher degree reflect that females of the current study had more complex repairs performed. In the same study, Kurian et al. concluded increasing mesh size as an independent risk factor for longer hospital stay, which is in line with our analysis, again reflecting increased dissection to lead to increased risk of overnight stay. This notion was also supported by the fact that the significant majority of patients who required overnight stay underwent a TAR procedure, and furthermore, of all TAR patients, the roboTAR/bilateral TAR procedure had a higher representation in the group of patients staying overnight, with the insignificance most likely explained by type-II error. For patients undergoing total hip arthroplasty, an increased duration of surgery was also significantly correlated with LOS [25].

Our study showed that patient-reported post-operative pain levels were low and not associated with prolonged hospital stay. A tendency toward increased pain after a TAR procedure was noted; however, there were too few observations to conclude on this. Regarding pain in the group who required overnight stay, the results showed slightly higher levels at rest compared to the patients discharged the same day of surgery; however, this trend was not conclusive in general. One aim of ERAS protocols is to decrease patient-reported pain, and the current results support that our ERAS protocol lives up to this aim [26, 27].

The patient-reported quality of life improved significantly for the entire cohort. However, we did find that patients undergoing incisional hernia repair did not seem to improve to the same degree as those undergoing primary ventral hernia repair. This may be explained by the previous surgical trauma to the abdominal wall, as well as the—most often—extended dissection in incisional compared to primary ventral hernia repair. The quality of life of patients undergoing robotic incisional hernia repair may improve further beyond the first 30 post-operative days, which we intend on examining in future studies.

ERAS pathways within abdominal surgery remain a field of complexity, and there are different opinions on whether the aim should be a general generic ERAS protocol applying to all patients, or if individualized protocols would be more appropriate [28–31]. This notion, and the fact that robotic surgery and ERAS protocols are rapidly evolving and gaining broader fields, highly approves the importance and relevance of this study.

A strength of this study is the prospective design, including the patient-reported outcomes, which we consider important in an analysis of a fast-track surgery setup. Optimal ERAS protocols for ventral hernia repair include weight loss for obese patients and complete smoking cessation. In the current study, patients with severe pain or intermittent bowel obstruction due to the hernia underwent surgery without weight loss or smoking cessation, which may have impacted on the results. We had no information about potential further analgesic requirement after the first three post-operative days and although opioids are optimally completely avoided in post-operative analgetic treatment, the protocol in the current study allowed only a limited amount of morphine, thus marginalizing the risk of subsequent opioid overuse. In the current study, we examined the outcomes of the total peri-operative care-bundle, including the described pre- and post-operative analgesic regimen, leaving any further need for analgesics as a potential limitation to our study.

## Conclusion

In the current study, an ERAS protocol after RVHR was associated with a high rate of outpatient procedures with low patient-reported pain scores. Risk factors of overnight stay in hospital include incisional hernia repair, TAR procedure, longer operating times, and larger mesh dimensions. Patients expected to carry these attributes should be given more attention pre-operatively, and future studies examining ERAS protocols in a robotic setting should be prospective with a focus on patient individualized pre-habilitation within this patient group.
